# The increased frequency of combined El Niño and positive IOD events since 1965s and its impacts on maritime continent hydroclimates

**DOI:** 10.1038/s41598-022-11663-1

**Published:** 2022-05-09

**Authors:** He-Ming Xiao, Min-Hui Lo, Jin-Yi Yu

**Affiliations:** 1 Department of Atmospheric Sciences, National Taiwan University, Taipei, Taiwan, ROC; 2 Department of Earth System Science, University of California, Irvine, CA USA

**Keywords:** Climate sciences, Hydrology

## Abstract

The Indian and Pacific Oceans surround the Maritime Continent (MC). Major modes of sea surface temperature variability in both oceans, including the Indian Ocean Dipole (IOD) and El Niño–Southern Oscillation (ENSO), can strongly affect precipitation on the MC. The prevalence of fires in the MC is closely associated with precipitation amount and terrestrial water storage in September and October. Precipitation and terrestrial water storage, which is a measurement of hydrological drought conditions, are significantly modulated by Indian Ocean Dipole (IOD) and El Niño events. We utilize long-term datasets to study the combined effects of ENSO and the IOD on MC precipitation during the past 100 years (1900–2019) and find that the reductions in MC precipitation and terrestrial water storage are more pronounced during years when El Niño and a positive phase of the IOD (pIOD) coincided. The combined negative effects are produced mainly through an enhanced reduction of upward motion over the MC. Coincident El Niño-pIOD events have occurred more frequently after 1965. However, climate models do not project a higher occurrence of coincident El Niño-pIOD events in a severely warming condition, implying that not the global warming but the natural variability might be the leading cause of this phenomenon.

## Introduction

Wildfire outbreaks occur worldwide, threatening lives and causing property damage. One of the most fire-prone places in the world is the Maritime Continent (MC), which is located in the deep tropics and flanked by the tropical Pacific Ocean to the east and the tropical Indian Ocean to the west. Farmers in the MC set fires to clear land for planting palm trees. Although they usually manage to clear lands safely, fires can get out of control in exceptionally dry years and cause catastrophe. In 2015, schools and offices in the MC faced closures and planes were unable to take off due to the toxic smog caused by severe wildfires. Studies have revealed that MC fires usually begin in the boreal summer and peak in September and October (SO)^1^ and that wildfires occur most frequently during years with reduced precipitation^2–5^ (Fig. S1). Due to its geographical location, MC precipitation is significantly influenced by both El Niño–Southern Oscillation (ENSO) events to the east and Indian Ocean Dipole (IOD) events to the west^6,7^.

ENSO is the leading mode of sea surface temperature (SST) variability in the Pacific and is characterized by warm SST anomalies in the equatorial eastern-to-central Pacific during its El Niño phase. El Niño events typically develop during boreal summer and mature during boreal winter. The first leading mode of SST variability in the Indian Ocean is the basin-wide monopole, and the IOD is the second leading mode statistically^8–10^. During its positive phase, the IOD (pIOD; hereafter) is characterized by positive SST anomalies in the western Indian Ocean and negative SST anomalies in the eastern Indian Ocean. Although the magnitude of SST anomalies associated with the IOD is typically smaller than those associated with El Niño events, the IOD is essential in modulating precipitation across the MC^1^. The pIOD events can affect the precipitation not only by inducing anomalous Walker subsidence but also by reducing water vapor transport into the MC from the Indian Ocean^10^. The consensus of previous studies is that both El Niño and the pIOD can reduce MC precipitation^6,11,12^, because their warm SST anomalies weaken the Pacific and Indian Ocean branch of the Walker circulation resulting in anomalous subsidence over the MC region. Previous studies have shown the combined role of the El Niño type and the IOD phase in affecting the fire activities over the MC regions from 1979 to 2016^11^. In addition, in the past several decades, fire events have been increased dramatically associated with more frequent droughts. Whether such frequent droughts are related to the occurrence of both El Niño and pIOD events has not yet been studied.

It is known well that the IOD can be externally forced by ENSO^13^ and internally produced by the Indian Ocean–atmosphere coupling^8,10,14–16^, and the ENSO can also be forced by IOD^15,17^. Typically, an El Niño event forces a pIOD event during its developing year via the oceanic channel^18^ and the atmospheric bridge mechanism^19–21^. Model experiments suggested that approximately one-third of IOD events can be explained by ENSO variability while the other two-thirds are internally generated^14,16^. It has also been increasingly recognized that ENSO properties have changed in recent decades, possibly due to global warming or natural decadal variability^22–24^. Different types of ENSO, such as the Eastern Pacific and Central Pacific types^25–27^, produce different impacts on the Walker circulation^28^. Also, the degrees of drought caused by ENSO get more severe over the past decades^29^. The ENSO-IOD relationship may have changed as a result of the change in ENSO properties and/or the internal atmosphere–ocean coupling in the Indian Ocean. It is, therefore, unknown whether (1) coincident El Niño-pIOD events have occurred more or less frequently during recent decades, and (2) the combined effects of coincident El Niño-pIOD events are similar or very different from the effect of El Niño-only and pIOD-only events. In this study, we utilize long-term (more than 100 years) observational datasets and model results, mainly focusing on September and October that is the mature time of IOD event and the strongest fire seasons to address these two questions. Notice that the MC area is defined as the region from 10° S to 10° N and 90° E to 150° E in this study.

## Results

**Figure 1:**
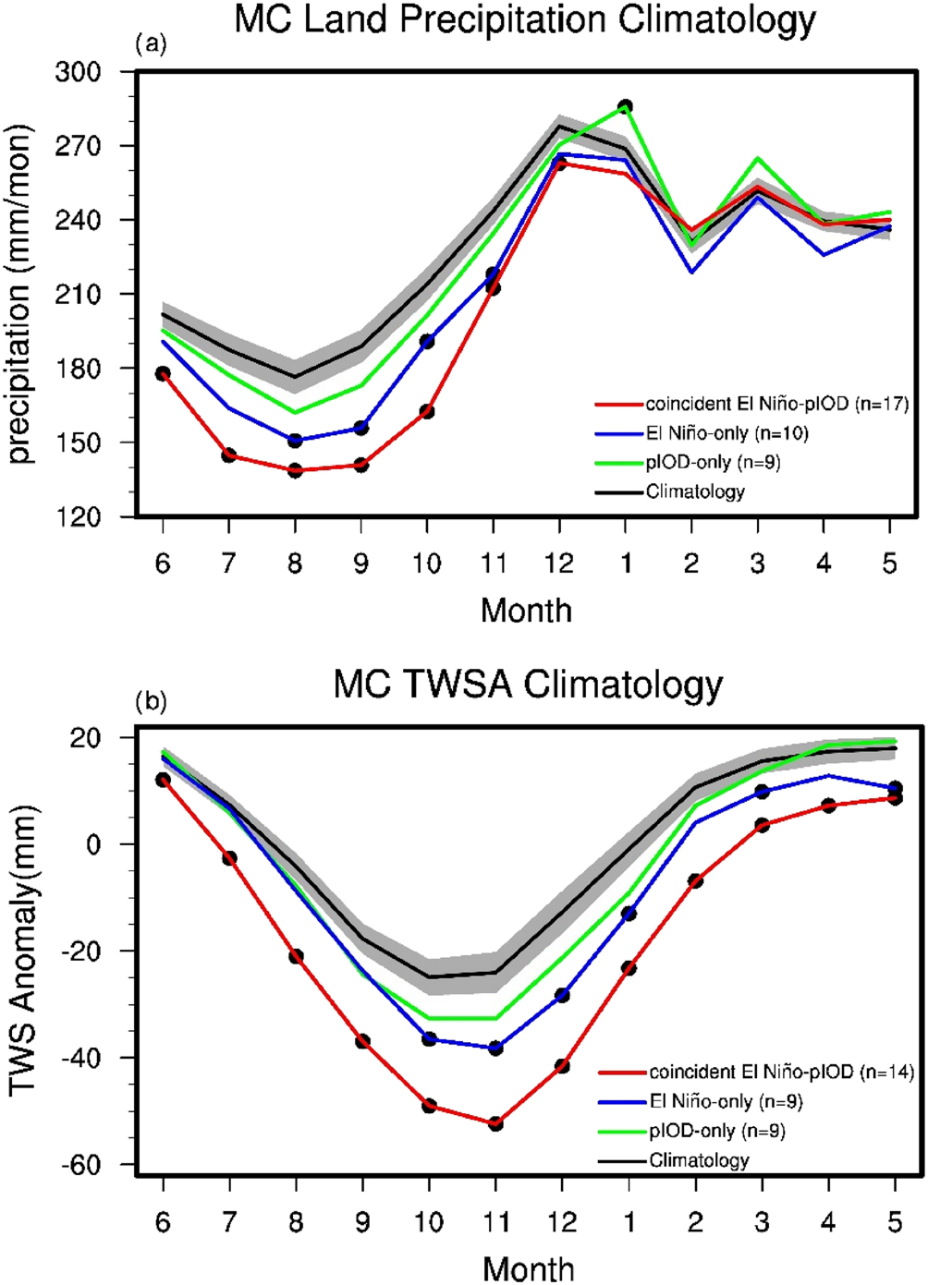
The black, green, blue, and red lines represent the annual cycle in (**a**) MC precipitation (from GPCC) averaged for the period 1900 to 2019 and (**b**) MC simulated monthly terrestrial water storage anomalies (TWSA) averaged from 1911 to 2015 for the climatology, pIOD-only, El Niño-only, and coincident El Niño-pIOD years, respectively. The gray shading indicates the 95% confidence interval and the dots represent significant differences between each group and the climatology for either precipitation or TWSA (*p* < 0.05).

Figure 1a shows the climatology annual cycle in MC precipitation, with the driest and wettest months observed in August and December, respectively (black line with grey shaded). The precipitation decreases gradually from December to August of the next year. The annual cycles calculated from the El Niño-only and pIOD-only years were similar to the climatological from December to May of the next year, and drier than the climatology from June to November. Moreover, the annual cycles of MC precipitation for the coincident El Niño-pIOD years were notably drier than the climatology from June to December. During coincident event years, MC precipitation decreased the most (up to 100 mm) during September and October (SO).

Studies have shown that wildfires are most likely to occur in the MC during SO^1,5^ (also seen Fig. S1) and that the fire-prone months usually lag the dry months because the ground cover requires approximately 1 to 3 months to transform into low water condition^30^. Furthermore, the land can remember atmospheric forcing conditions and responds several months later^31^. This lagged response feature can be clearly seen in Fig. 1b, which presents the annual cycle of the TWS anomaly (remove annual mean, TWSA, hereafter) in the MC from the CLM. The figure reveals that the lowest TWSA occurs about 2 months after the driest precipitation month. Additionally, similar to the precipitation response in the pIOD-only years, insignificantly decreases in TWSA were observed compared to the climatology from September to next January. The TWSA in the El Niño-only years was slightly lower than that in the pIOD-only years. The TWSA in the coincident El Niño-pIOD years was significantly lower than the climatology throughout the year. The differences in TWSA in the coincident years were particularly evident from June onwards.

The signal of pIOD and El Niño events starts from boreal summer; then, MC precipitation was deficient; consequently, the TWSA gradually decreased and reached its minimum in November, two months after the least precipitation month. As mentioned, because of the reduced precipitation and lower TWS in SO, the ground cover becomes dry and serves as potential fuel for fire events. Thus, this study focuses on the SO period, which is the peak of fire season, and explores why so much less precipitation occurs during coincident El Niño-pIOD events than during pIOD-only or El Niño-only events.

**Figure 2:**
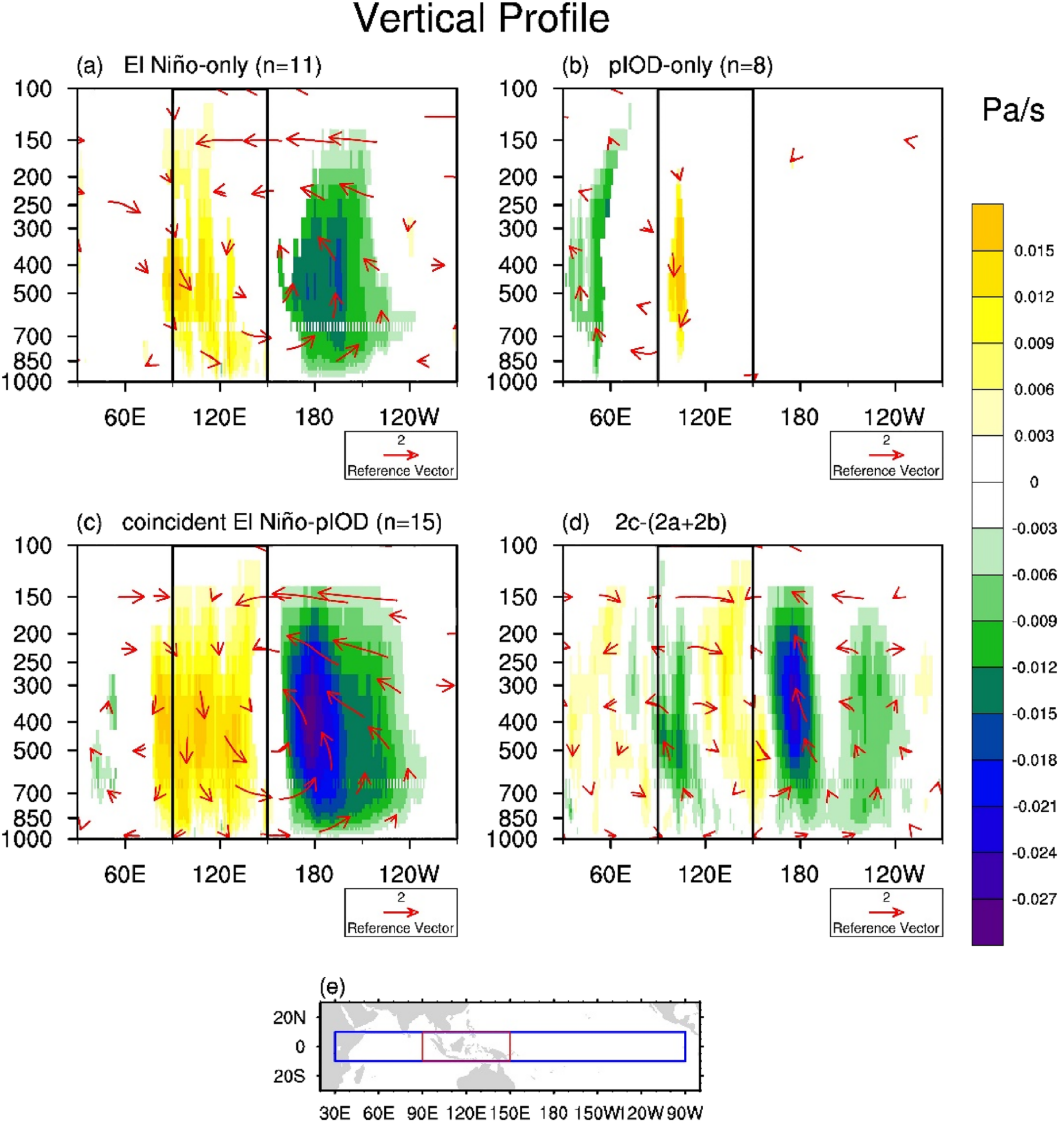
Composites of wind (vector, m/s) and vertical velocity (shading, Pa/s) anomalies during SO for (**a**) El Niño-only, (**b**) pIOD-only, (**c**) coincident El Niño-pIOD events, and (**d**) difference between coincident events (**c**) and the sum of El Niño-only and pIOD-only (**a**,**b**), or (**c**)-(**a**,**b**). (**e**) Map of the selected area (blue box) for the meridional average. When the composite of the wind was calculated, the vertical velocity was multiplied by − 100. The black boxes in (**a**–**d**) represent the MC area including the ocean and averaged meridional from − 10° to 10°N. Only significant areas were plotted (p < 0.05) in panels (**a**–**d**). The box outlined in red in panel (**e**) is the study domain of the Maritime Continent used. The figures were plotted by NCAR Command Language (NCL) version 6.5.0 (https://www.ncl.ucar.edu/). The figures were plotted by NCAR Command Language (NCL) version 6.5.0 (https://www.ncl.ucar.edu/).

Figure 2 shows the vertical cross-section of the vertical velocity over the equatorial Indo-Pacific Oceans (averaged between 10° S and 10° N) during SO. Compared to the climatology, weak and narrow areas of sinking motion were observed over the MC area during El Niño-only and pIOD-only events. During pIOD-only events, for example, anomalous subsidence occurred between 850 and 200 hPa over a small area near 105° E. However, the subsidence anomaly is broad and covers the entire MC during the coincident El Niño-pIOD events, in which the anomalous weak Walker circulation over the equatorial Pacific was also more robust than that of the El Niño-only events. Thus, the coincident El Niño-pIOD events on average produced stronger anomalous subsidence over the MC area associated with a greater reduction in precipitation than El Niño-only and pIOD-only events. We also examined whether the anomalies produced by the coincident events are equal to or greater than the combined effects produced by the El Niño-only and pIOD-only events. Figure 2d shows the difference between the coincident events (Fig. 2c) and the summation of El Niño-only and pIOD-only (Fig. 2a,b). This difference clearly shows strong subsidence anomalies over the MC regions. Therefore, the impacts produced by the coincident events are not just a linear combination but the manifestation of a nonlinear amplification of the individual impacts from the El Niño-only and pIOD-only events. This nonlinear amplification may be due to the nonlinear relationship between SST and convection. Another possible cause is the influence of the IOD events on the ENSO events, that is the strength of El Niño in the coincident El Niño-pIOD events usually stronger than the El Niño-only events^17^.

**Figure 3:**
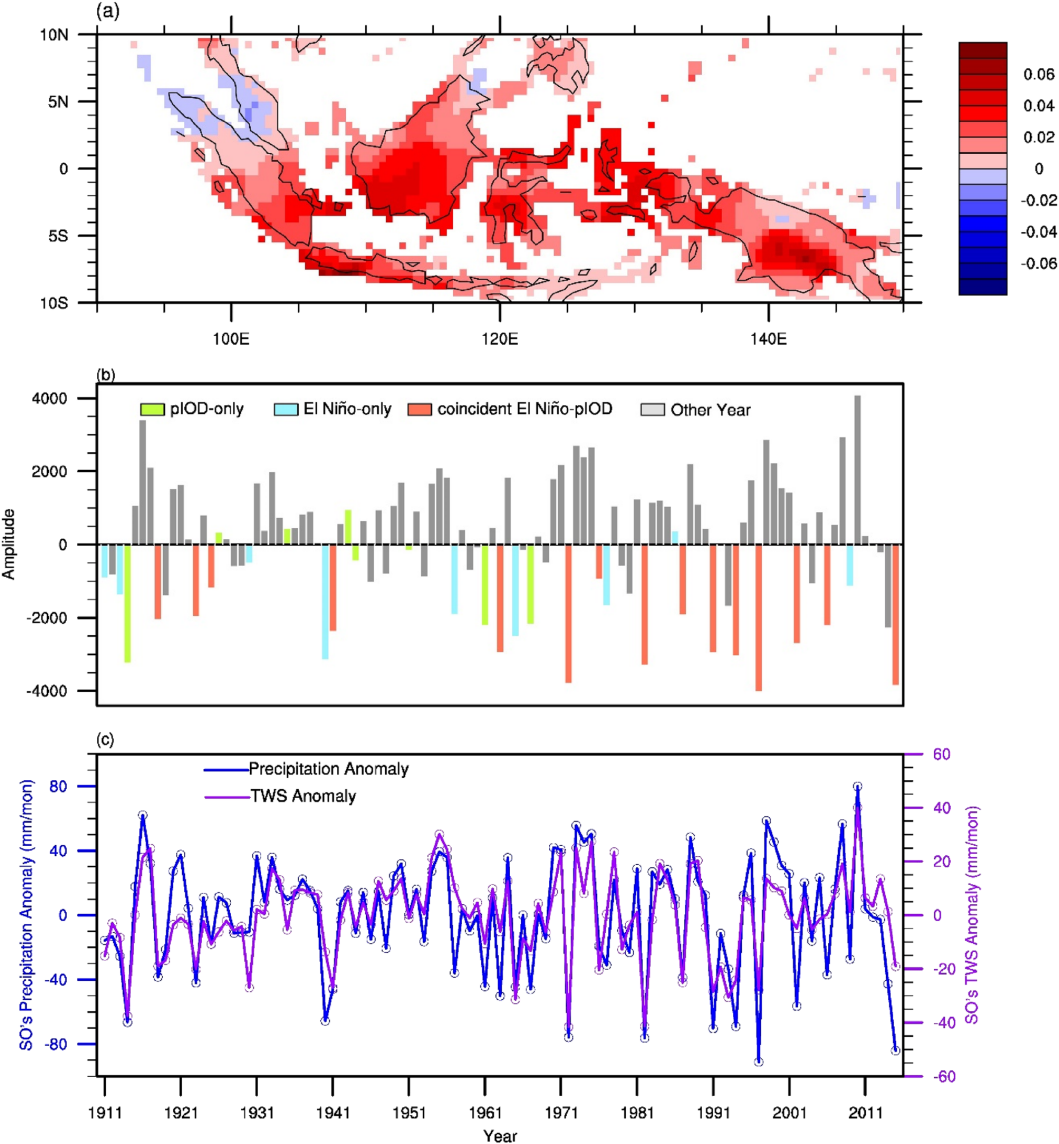
(**a**) First EOF mode of observed MC SO precipitation for the period 1911–2015. This mode explains approximately 34.2% of the variance. (**b**) First principal component (bar) during SO. The bars are rendered in green, blue, red, and gray for the pIOD-only, El Niño-only, coincident El Niño-pIOD, and other years, respectively. (**c**) The precipitation anomalies (blue line) and TWS anomalies (purple line) in SO. The anomalies are computed by removing annual cycle. The figures were plotted by NCAR Command Language (NCL) version 6.5.0 (https://www.ncl.ucar.edu/).

**Figure 4:**
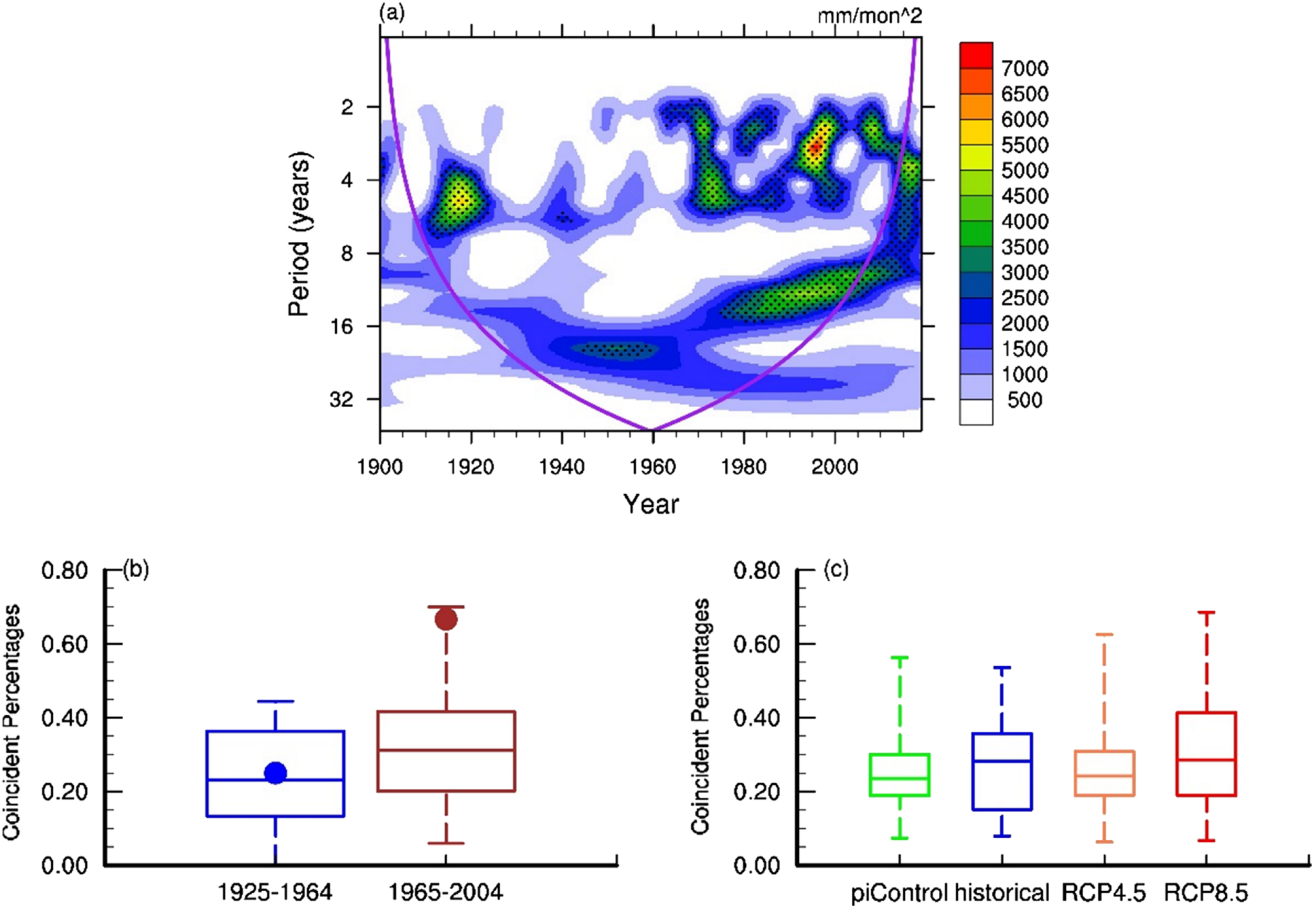
(**a**) Wavelet power spectra of MC precipitation during SO, areas where the 95% confidence interval is exceeded are stippled. (**b**) Box plot of the coincident El Niño-pIOD percentages from 18 CMIP5 historical simulations for two periods (1925–1964 vs 1965–2004). The coincident percentage is defined as the ratio of the number of coincident events to the sum of El Niño-only events, pIOD-only events, and coincident El Niño-pIOD events for each model. The results from observations were dotted in blue and red, respectively. (**c**) same as panel (**b**), but for the 18 CMIP5 piControl, historical, RCP4.5 and RCP8.5 simulations. The figures were plotted by NCAR Command Language (NCL) version 6.5.0 (https://www.ncl.ucar.edu/).

The characteristics of the El Niño have changed since the late twentieth century^22,23,32,33^. To explore the temporal variations of MC’s hydroclimate during the twentieth century, an empirical orthogonal function (EOF) analysis was applied to the SO precipitation for the period 1911 to 2015. The first EOF mode explains approximately one-third (34.2%) of the total variance in the precipitation (Fig. 3a,b). This leading EOF mode (in its positive phase) is characterized by a uniformly negative anomalous precipitation pattern through the MC except over the northern part of Sumatra and the western part of Malaysia (Fig. 3a). Figure 3b displays the principal component (PC) of this leading mode. In the figure, pIOD-only, El Niño-only, coincident El Niño-pIOD, and neutral years are indicated by green, blue, red, and gray bars, respectively. The anomalous precipitation time series is also shown in Fig. 3b, and has a characteristic similar to those of the 1st PC (Fig. 3b). In Fig. 3b, the number of red bars increased slightly during around 1920 and increased dramatically after 1965, indicates that the frequency of coincident El Niño-pIOD events has increased in these two periods. Also, the amplitude of red bar is larger after 1965. At the same time, the precipitation anomalies and TWSA in SO decreased a lot after 1965 (Fig. 3c), implies that the extend of fires may be more severe after 1965. A wavelet analysis of the SO anomalies in the MC precipitation (Fig. 4a) further reveals this high-frequency variation in the precipitation with a period of about 2–4 years. This variation was strong during 1910–1930, weakened afterward, but intensified again beginning around 1965. These two strong periods coincide with the period when the coincident El Niño-pIOD events occurred most frequently at around 1920 and during the past 40 years (Figs. 3b and 4a).

We next use 18 CMIP5 historical simulations to further explore the occurrence frequency of the coincident events (Fig. 4b). The coincident percentage is defined as the ratio of the number of coincident events to the sum of El Niño-only events, pIOD-only events, and coincident El Niño-pIOD events in each model simulation. The mean and standard deviation of the coincident percentages during 1905–2004 across the 18 models are displayed in Fig. 4b. We plotted coincident percentage of 1925–1964 and 1965–2004, the two periods with two relatively less and more coincident El Niño-pIOD events from observations (Fig. 3b). Consistent with the findings in observations, model simulations suggest that coincident El Niño-pIOD events are likely to occur more frequently during the period 1965–2004 than during 1925–1964, although the coincident percentage in 1965–2004 is greatly lower than the same period in observation. We also use CMIP5 projections to explore possible future changes in the frequency of coincident events. Figure 4c shows the mean value and standard deviation of the coincident percentages in the preindustrial control (piControl), representative concentration pathway 4.5 (RCP4.5, from 2006 to 2099) and representative concentration pathway 8.5 (RCP8.5, from 2006 to 2099) simulations from the 18 CMIP5 models. The figure shows that the coincident percentage median increased from piControl to historical, decreased from historical to RCP4.5, and increased again from RCP4.5 to RCP8.5. The fact that the percentages difference for these four simulations (Fig. 4c) are too small prevents us from attributing the shift in the occurrence of coincidental ENSO and IOD events to global warming.

## Discussion and conclusions

This study employed statistical analysis of observations, reanalysis datasets, and climate model simulations to better understand how dry season precipitation over the MC area is affected by coincident El Niño-pIOD events during 1900–2019. Our results indicated that coincident El Niño-pIOD events produce a much stronger suppression effect on MC precipitation from June to November than El Niño-only or pIDO-only events. A lagged response in TWS, in turn, leads to drier ground cover and increases the risk of severe fires in September–October. In other words, it is the significant decrease of precipitation in coincident El Niño-pIOD events and the lagged response of total water storage, the severe fires happened in SO (Fig. S1). We also discovered that the occurrence frequency of the coincident El Niño-pIOD events has greatly increased since the mid-1960s in the observations (Fig. 3b) and CMIP5 historical simulations (Fig. 4b).

Previous studies showed that the relationship between ENSO and IOD has inter-decadal variations, and the strengths of ENSO are also highly correlated with IOD^34,35^. Compared to global warming, the variation of coincident El Niño-pIOD events number in the past 120-year might be more associated with natural variability. This multi-decadal change of relationship between ENSO and IOD can also be seen from MC’s SO land precipitation (Fig. 4a). In fact, the relationship between the strength of ENSO and the strength of IOD also has multi-decadal variations (Fig. S2). A series of numerical experiments can be utilized to understand the factors (such as global warming^15,33,36^, natural decadal variabilities^22,23,37^, anthropogenic land use changes^38^) controlling the multi-decadal change of the relationship between ENSO and IOD, which will be critical for understanding the fire occurrence frequency in this region in the future. Except for the impacts of coincident El Niño-pIOD events, the recent 2019 drought is caused by an extreme pIOD-only event. The 2019 extreme pIOD-only event is associated with global warming^39^, implying the importance of future pIOD events.

## Datasets and methods

The precipitation datasets used were sourced from the 0.5° × 0.5° Global Precipitation Climatology Centre (GPCC) Full Data Monthly Analysis Version 2020^40^, which was constructed from ground observations, the 1° × 1° Hadley Centre Sea Ice and Sea Surface Temperature dataset version 1 (HadISST1)^41^, the 0.25° × 0.25° Global Fire Emissions Database version 4 (GFEDv4s)^42^ and the 1.25° × 1.25° Twentieth Century Reanalysis dataset^43^ from the European Centre for Medium-Range Weather Forecasts. We select the period 1900 to 2019 to examine precipitation and SST and the period 1900 to 2010 for the atmospheric circulation analysis. Another monthly gridded precipitation dataset from the Climatic Research Unit (CRU)^44^, and the fifth version of Extended Reconstructed Sea Surface Temperature (ERSSTv5)^45^ were also examined. The details of these reanalysis datasets are listed in Table 1. To understand the degree of drought during different years, we calculated terrestrial water storage (TWS) changes during the study period. We examined simulated TWS from the Community Land Model (CLM5.0)^46^, which was forced with atmospheric observations. The experiment period was 1900 to 2015, and simulations of the last 105 years—that is, from 1911 to 2015—were utilized. To explore whether global warming might have a substantial impact on precipitation variations in the MC in the future, we examined the results from 18 models (listed in Table 2) in the Coupled Model Intercomparison Project—Phase 5 (CMIP5).

**Table 1:** The details of selected reanalysis datasets.

Variable	Organization	Version	Available time	Selected time	Resolution
**Precipitation**	**Global Precipitation Climatology Centre**	**GPCC Full Data Monthly Version 2020**	**1891–2019**	**1900–2019**	**0.5° × 0.5°**
	Climatic Research Unit	CRU TS4.03 Precipitation	1901–2018	1901–2018	0.5° × 0.5°
**SST**	**Met Office Hadley Centre**	**HadISST1**	**1870-present**	**1900–2019**	**1.0° × 1.0°**
	National Oceanic and Atmospheric Administration	ERSST V5	1854–present	1900–2019	2.0° × 2.0°
**Wind**	**European Centre for Medium-Range Weather Forecasts**	**ERA-20C**	**1900–2010**	**1900–2010**	**1.25° × 1.25°**
**Fire**	**Global Fire Emissions Database, Version 4.1**	**GFEDv4**	**1997–present**	**1997–2021**	**0.25° × 0.25°**

These datasets used in figures are in bold.

**Table 2:** The 18 CMIP5 piControl, historical, RCP4.5 and RCP8.5 coupled models list.

	Model ID	Institution
1	ACCESS1-0	CSIRO and BOM
2	CanESM2	Canadian Centre for Climate Modelling and Analysis
3	CCSM4	National Center for Atmospheric Research
4	CESM1-BGC	National Science Foundation, Department of Energy, National Center for Atmospheric Research
5	CNRM-CM5	Centre National de Recherches Meteorologiques / Centre Europeen de Recherche et Formation Avancees en Calcul Scientifique
6	GFDL-CM3	Geophysical Fluid Dynamics Laboratory
7	GFDL-ESM2G	Geophysical Fluid Dynamics Laboratory
8	GISS-E2-H	NASA Goddard Institute for Space Studies
9	GISS-E2-R	NASA Goddard Institute for Space Studies
10	HadGEM2-AO	National Institute of Meteorological Research/Korea Meteorological Administration
11	INM-CM4	Institute for Numerical Mathematics
12	IPSL-CM5A-LR	Institut Pierre-Simon Laplace
13	IPSL-CM5A-MR	Institut Pierre-Simon Laplace
14	MIROC5	Atmosphere and Ocean Research Institute (The University of Tokyo), National Institute for Environmental Studies, and Japan Agency for Marine-Earth Science and Technology
15	MIROC-ESM	Japan Agency for Marine-Earth Science and Technology, Atmosphere and Ocean Research Institute (The University of Tokyo), and National Institute for Environmental Studies
16	MPI-ESM-LR	Max Planck Institute for Meteorology
17	MPI-CGCM3	Max Planck Institute for Meteorology
18	NorESM1-M	Norwegian Climate Centre

Long-term linear trends and annual cycles were removed from the original datasets. We use the dipole mode index (DMI)^10^ and Niño3.4 index^47^ to represent IOD and ENSO activities, respectively. A pIOD event was identified when the mean values of the IOD index exceed one standard deviation for the June-July–August (JJA) or September–October-November (SON) season. An ENSO event was identified when the mean value of Niño3.4 SST exceeds one standard deviation for the November–December-January season. When an El Niño event and a pIOD event occur during the same year, they are referred to as a coincident El Niño-pIOD event. Conversely, El Niño-only and pIOD-only events refer, respectively, to an El Niño event occurring without an IOD event and a pIOD event occurring without an ENSO event. Table 3 shows the years of the pIOD-only, El Niño-only, and coincident El Niño-pIOD events during 1900–2019.

**Table 3:** The year of El Niño-only, pIOD-only, and coincident El Niño-pIOD events during 1900–2019.

El Niño-only	1905, 1911, 1913, 1930, 1940, 1957, 1965, 1977, 1986, 2009
pIOD-only	1914, 1926, 1935, 1943, 1944, 1951, 1961, 1967, 2012, 2019
coincident El Niño-pIOD	1902, 1918, 1923, 1925, 1941, 1963, 1972, 1976, 1982, 1987, 1991, 1994, 1997, 2002, 2006, 2015

## Supplementary Information

Supplementary Information.

## Data Availability

Those data used in this study can be obtained from GPCC: https://opendata.dwd.de/climate_environment/GPCC/html/download_gate.html; CRU https://crudata.uea.ac.uk/cru/data/hrg/ The HadISST data are available from https://www.metoffice.gov.uk/hadobs/hadisst/ The ERSST data are available from https://www.ncei.noaa.gov/access/metadata/landing-page/bin/iso?id=gov.noaa.ncdc:C00927. The ERA20C are available from https://www.ecmwf.int/en/forecasts/datasets/reanalysis-datasets/era-20c. The GFEDv4 data are available from https://daac.ornl.gov/VEGETATION/guides/fire_emissions_v4_R1.html. All the data processing and figures code can be downloaded in the Github (https://github.com/hm-xiao/Combined-Effects-of-ENSO-and-IOD-on-the-Maritime-Continent-s-Land-Precipitation-During-SO-code/tree/main).
