# Supplementary material for: The increased frequency of combined El Niño and positive IOD events since 1965s and its impacts on maritime continent hydroclimates
Source: Sci Rep. 2022 May 9;12:7532. doi: 10.1038/s41598-022-11663-1 (PMC9085806; doi:10.1038/s41598-022-11663-1)
Supplement: Supplementary file 1 — Supplementary Information. [file 41598_2022_11663_MOESM1_ESM.docx]

The Increased Frequency of Combined El Niño and Positive IOD events since 1965s and Its Impacts on Maritime Continent Hydroclimate

He-Ming Xiao^1^, ^*^Min-Hui Lo^1^, Jin-Yi Yu^2^

^1^He-Ming Xiao ([xhm3219057@gmail.com](mailto:xhm3219057@gmail.com)),

^1^Min-Hui Lo ([minhuilo@ntu.edu.tw](mailto:minhuilo@ntu.edu.tw)),

^2^Jin-Yi Yu ([jyyu@uci.edu](mailto:jyyu@uci.edu))

^1^Department of Atmospheric Sciences, National Taiwan University, Taipei, Taiwan

^2^Department of Earth System Science University of California, Irvine, CA, USA

^*^corresponding author: Min-Hui Lo ([minhuilo@ntu.edu.tw](mailto:minhuilo@ntu.edu.tw))


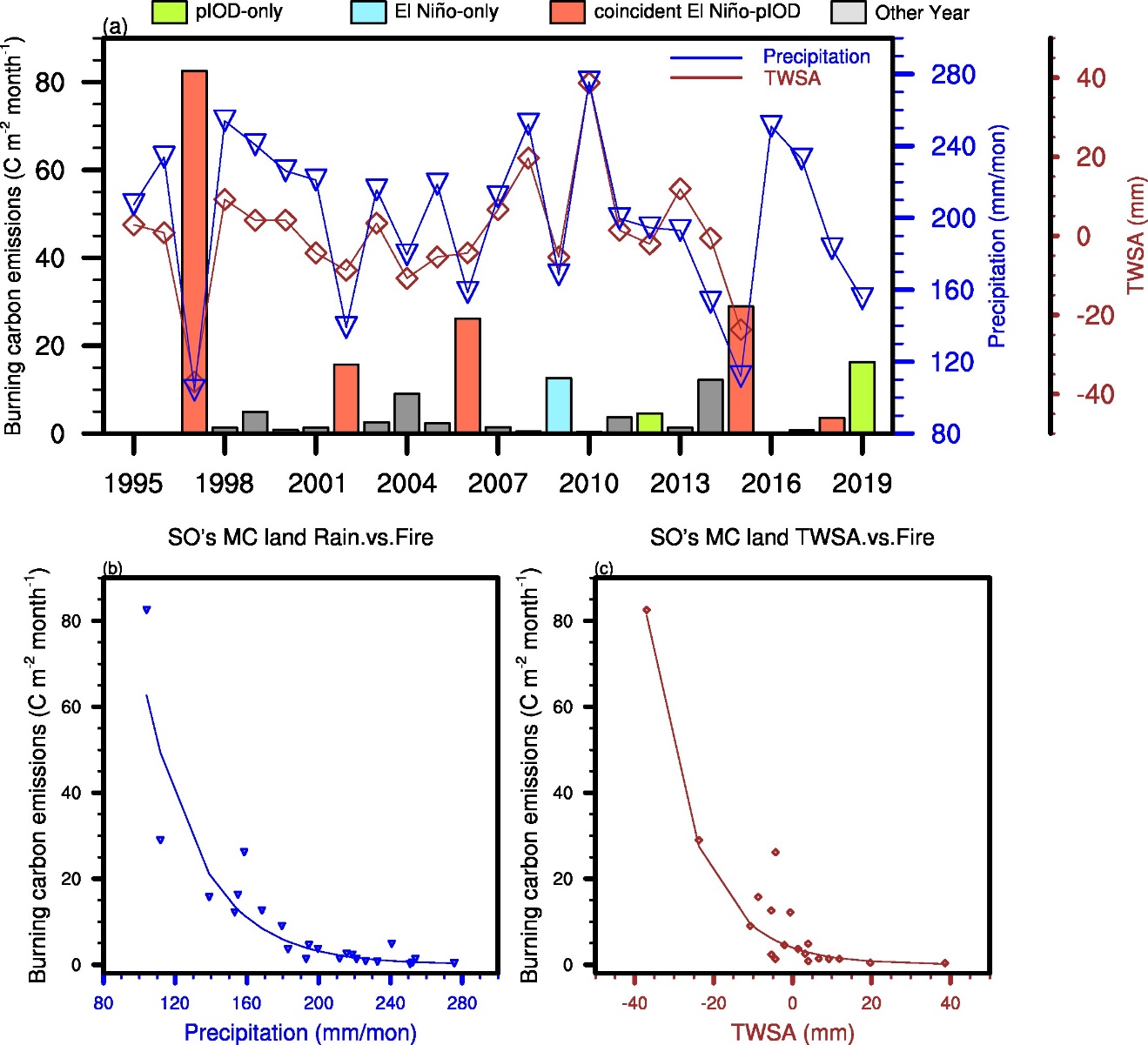


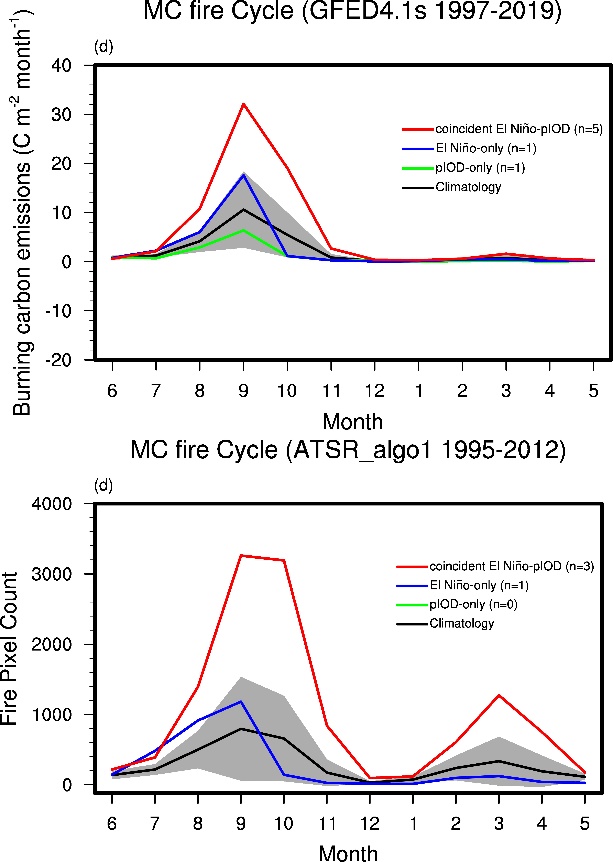


Figure S1. Fires (Burning carbon emission, C m^-2^ month^-1^). The bars are rendered in green, blue, red, and gray for the pIOD-only, El Niño-only, coincident El Niño-pIOD, and other years, respectively. The black, and brown line represents the MC precipitation (from GPCC) and MC simulated TWSA during SO. (b) The distribution (dot) and exponential relationship (line) of SO’s fires and precipitation. (c) same as (b), but for the TWSA. (d) The black, green, blue, and red lines represent the fire’s annual cycle averaged from 1997 to 2019 for the climatology, pIOD-only, El Niño-only, and coincident El Niño-pIOD years, respectively. The gray shading indicates the 95% confidence interval. The figures were plotted by NCAR Command Language (NCL) version 6.5.0 (<https://www.ncl.ucar.edu/>).


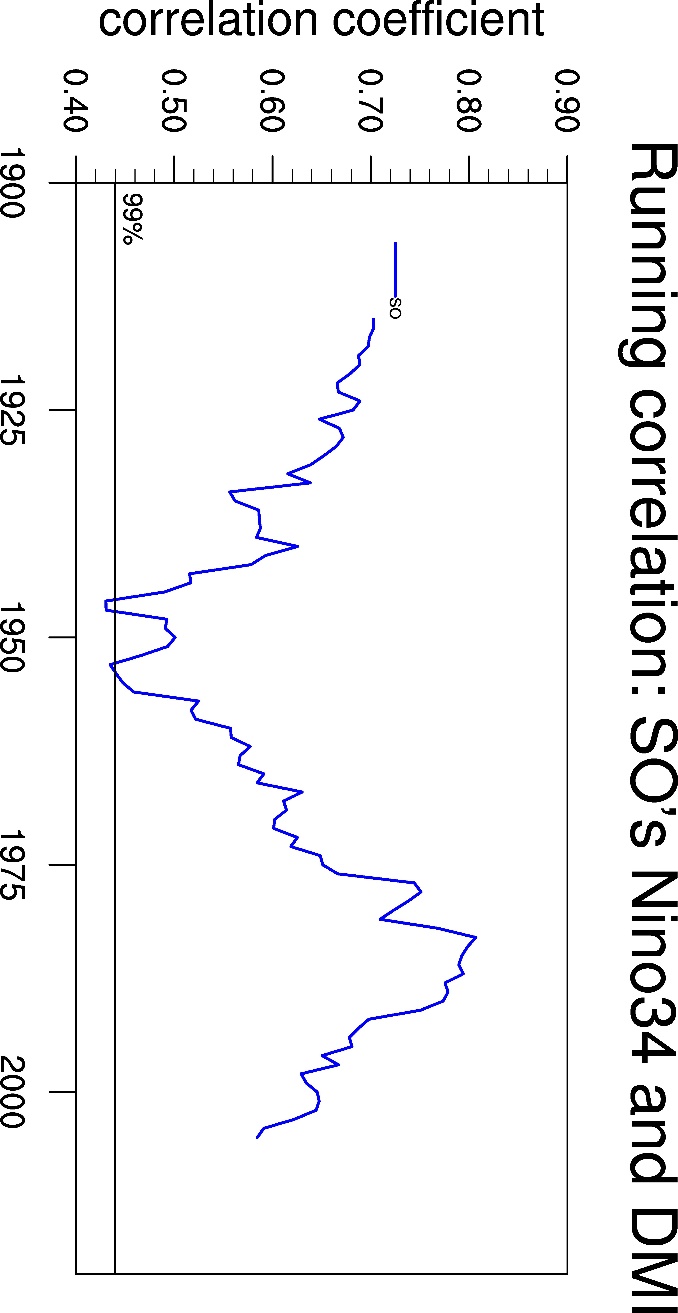


Figure S2. 31-year running correlation between Nino34 and DMI in SO (blue line). The result was shown in the central year. For example, we calculate 1901-1931 SO’s Nino34 and DMI correlation, showing the result in 1916. The figures were plotted by NCAR Command Language (NCL) version 6.5.0 (<https://www.ncl.ucar.edu/>).
